# Effect of in-home and community-based services on the functional status of elderly in the long-term care insurance system in Japan

**DOI:** 10.1186/1472-6963-12-239

**Published:** 2012-08-04

**Authors:** Pedro Olivares-Tirado, Nanako Tamiya, Masayo Kashiwagi

**Affiliations:** 1 Department of Health Services Research, Graduate School of Comprehensive Human Sciences, University of Tsukuba, 1-1-1 Tenno-dai Tsukuba, Ibaraki, 305-8575, Japan

## Abstract

**Background:**

Japan is setting the pace among aging societies of the world. In 2005, Japan became the country with the highest proportion of elderly persons in the world. To deal with the accelerated ageing population and with an increased demand for long-term care services, in April 2000 the Japanese government introduced a mandatory social Long-Term Care Insurance System (LTCI), making long-term care services a universal entitlement for elderly. Overseas literature suggests that the effectiveness of a home visiting program is uncertain in terms of preventing a decline in the functional status of elderly individuals. In Japan, many studies regarding factors associated with LTC service utilization have been conducted, however, limited evidence about the effect of LTC services on the progression of recipient disability is available.

**Methods:**

Data were obtained from databases of the LTC insurer of City A. To examine the effect of in-home and community-based services on disability status of recipients, a survival analysis in a cohort of moderately disabled elderly people, was conducted.

**Results:**

The mean age of participants was 81 years old, and females represented 69% of the participants. A decline or an improvement in functional status, was observed in 43% and 27% of the sample, respectively. After controlling for other variables, women had a significantly greater probability of improving their functional status during all phases of the observation period. The use of “one service” and the amount of services utilized (days/month), were marginally (p = < 0.10) associated with a greater probability of improving their functional status at 12 months into the observation period.

**Conclusions:**

The observed effects of in-home and community-based services on disability transition status were considered fairly modest and weak, in terms of their ability to improve or to prevent a decline in functional status. We suggest two mechanisms to explain these findings. First, disability transition as a measure of disability progression may not be specific enough to assess changes in functional status of LTCI recipients. Secondly, in-home and community-based services provided in City A, may be inappropriate in terms of intensity, duration or quality of care.

## Background

In 1990, the percentage of people aged 65 years and older in Japan was 12% [1,2]. Since then, the Japanese population has aged rapidly. In 2006, the Japanese population reached 127.7 million, and the proportion of elderly was 20.8%, the highest in the world. By the year 2025, the proportion of elderly is expected to reach 30% of the total population in Japan [3,4].

Considering the accelerated aging population and mainly, the increased needs for nursing care among the elderly population, the Japanese government introduced a long-term care insurance system (hereafter, LTCI) in April 2000 [3,5,6]. The purposes were to prevent a decline in functional status, to allow elderly people to live independently in the community for as long as possible, and to expand community-based care [7-12].

The LTCI system in Japan relies on a mandatory social insurance model, financed partially by general taxes, social contributions, and cost-sharing (co-payment). Universal benefits entitlement for elderly people is based strictly on the extent of their physical or mental disability. Local governments act as insurers and manage LTCI based on national guidelines. However, main issues such as certification of level of eligibility, insurance coverage, or fees for LTC services are all set uniformly across the country by the central government [5,6,13,14].

Certification of eligibility and corresponding benefit limits are based on a nationally standardized assessment process. Eligibility levels are primarily determined by a computerized algorithm based on current physical and mental status [8]. The final decision is made by a local expert committee after considering the medical report, but independent of the availability of any potential informal caregiver network and of the individual's income [8,15]. The eligibility decision i.e., the level of assistance/care needed and the monthly benefit limit, is then communicated to the applicant within 30 days of application [15]. Six eligibility levels were established when the LTCI system began; however, since 2005 LTCI reform, there are now seven eligibility levels; the two lowest levels are called “assistance required” (“*yo-shien*”), and the remaining five levels are called “care required” (“*yo-kaigo*”) [9,16,17]. Among the elderly certified as “care required”, those who are in less care needs, are defined as Care Level 1(CL1).

Theoretically, users are free to choose services, but care managers certified by prefectures actually make care plans based on the applicant’s certified assistance/care needs level, living environment, and requests from the user and family. Then, a care plan is designed, and the process concludes with a contract between a care-provider firm and the user. However, beneficiaries are re-evaluated every 6 months, and they may request changes to the care plan and may change the manager and/or provider if they are dissatisfied [5,9,16].

Only services, not cash benefits, are provided in the Japanese LTCI system. Those certified in the “*yo-shien*” category can only use community care or preventive services to help them lead self-supporting lives while maintaining their present physical condition as long as possible. Those certified in the “*yo-kaigo*” category receive home-based, community-based, or institutional care services [2,9].

The main categories of at-home care services include home-visit care, home-visit nursing, home-visit bathing service, home-visit rehabilitation, management guidance for in-home care, and rental service for assistive devices. Commuting services in Japan are defined as services delivered in a community-based facility, where users commute to receive personal care, support for activities of daily living, and physical exercises, and they return home the same day [6].

Many studies related to factors associated with LTC service utilization have been conducted in Japan [5,7,10,18,19]. However, little evidence is available about the effect of these services on the progression of disability in current users [20-22]. Moreover, overseas literature suggests that the effectiveness of home visiting programs remains controversial in terms of their preventing a decline in the functional status of elderly people [23-26].

The aim of this study was to examine the effect of in-home and community-based services on disability transition status in a cohort of elderly who were newly certified for Care Level 1(CL1) in a suburban city of Tokyo. Disability transition status represents a change in the functional status of the current LTCI system users as a consequence of a periodic re-evaluation conducted by insurers to determine changes in care needs level of beneficiaries. The instrument to evaluate these changes is the same used to decide the initial eligibility level of the applicants. Currently, is calculated as the difference in eligibility levels in the course of a year. We focused on subjects certified as CL1 to identify those truly in need of care because we assumed they are moderately disabled and consequently have the possibility of maintaining or improving their functional status by using the current services delivered by the LTCI system. A survival analysis was conducted to examine the effect of in-home-based and community-based services on disability transition status.

## Methods

### Data & participants

Participants were selected from the dataset of the LTC insurer of City A, located in a suburban area approximately 100 km west of Tokyo. The name of the city remain anonymous because this issue is defined in the contract with City A as a condition to use the database. The estimated population as of October 1 2006, was 52,343, and the proportion of elderly persons was 20.8% [11], the same as the average in Japan [1].

The database contained basic demographic characteristics and information on the utilization of insurance benefits and services, which is periodically collected by the insurer from LTC providers. Consent for use of the dataset was granted by the municipal government of City A after a formal application, along with an explicit pledge to protect the confidentiality of the data supplied. Ethical considerations were examined in accordance with Japanese epidemiological guidelines for secondary data analysis. Ethics approval was obtained from the Ethics Committee of the University of Tsukuba, Japan.

Participants in the study were selected based on the following criteria: (a) elderly persons i.e., aged 65 years or over; (b) newly certified as being eligible for CL1 benefits; (c) have used LTC services consecutively at least 6 or more months; and (d) have remained at least 3 months consecutively in CL1. We selected 6 months as a minimum stay in the LTCI system and 3 months in the CL1 to assure model stability. Participants who used facility services during the observation period were excluded (*n* = 20).

We started enrolling participants in July 2001 and continued for 57 months until March 2006, with an additional 15 months of follow-up until June 2007. The final data set contained records for 369 participants. This cohort was followed during their stay in the LTCI system to determine individual disability transition status every 6 months during the first 2 years and annually thereafter.

### Measurements

The disability progression of the elderly in Japanese LTCI system, is carry on by a periodic (every 6-months) re-evaluation of care needs level process, including ADL’s and IADL’s assessments, but also contains psychological, cognition, behavioral and medical dimensions. This evaluation process obey to a national standardized guidelines, is a complex and adequate process to evaluate care needs, but also is used to adjust the care plan and to decide changes of care manager and/or provider.

Certainly, the change in care needs level, it is not specific enough to asses fine changes in functional status of recipients however, as participants in this study are moderately disabled (CL1) we assumes that the change in care need level reflex a change in functional status rather than changes in the other dimension. Furthermore, in official and most of the scholar LTC Japanese literature, the change in care needs level of current users along with the time in LTCI system, also named as “disability transition status” is already accepted as a valid outcome regarding to functional status in Japan [20-22,27].

#### Dependent variable

The outcome variable for the analysis was the length of stay at CL1, defined as the total number of months at CL1, was calculated from the time when participants became LTC service users until a change in care-needs level category or censure. A participant who changed from the certified baseline CL1 was considered an event. Participants who dropped out of the LTCI system (*n* = 34), those were away from the system for more than 1 month (*n* = 57) or who remained at CL1 without having experienced an event during the observation period (*n* = 21) were treated as a censored observations.

#### Independent variables

Japanese LTCI system considers a multi-variety of services under in-home care and community-based services categories. The heterogeneity of interventions obey mainly to the demand of a comprehensive pool of services driving basically by care needs level of the current users. On the other hand, the ‘quasi-market” operating on side of the provision, strictly regulated by central government, trends to ensure uniformity and homogeneity in types, intensity and quality of long-term care services delivered according care needs level nationwide.

In our data a broad dispersion on the pattern of LTC services across strata was observed when LTC services are classified using the specific distribution proposed by the MHLW ( see, Additional file 1: Annex 1). The effect of the dispersion of services (just few cases by each category) strongly difficult any statistical analysis for the most of the services involved in the care of this cohort. Because, our goal was to examine the effect of some dimensions of the care-mix (type, quantity and intensity) of LTC services used on the length-of-stay in the initial certified care need level (CL1) and not oriented to examine the effect of each services or a specific mix of services, on our outcome.

Beyond the heterogeneity of the services and the “structural” limitations-availability of a sample large enough to evaluate specific services- the aggregation of the services under in-home care and community-based services, participants in both categories were allocated in a consistent, independent and mutually exclusive way. Thus, our analysis examine the effect of in-home services ‘family” versus commuting services ’family” on the length-of-stay in CL1, along an observation period of 36-months.This categorization also permit to include subjects using 2 or more services under the same “family”. Undoubtedly, this aggregate approach involved a trade-off between specificity and reliability. We opted by reliability”.

Three dimensions of the LTC services utilized, including type of service, number of different types of services, and services delivered (days/month) were examined as potential predictors for length of stay at CL1. Age, gender, income level, length of stay in the LTCI system, and utilization rate of insurance benefits were included in the overall model as potential confounders.

To evaluate types of service, we categorized services into three groups, in-home services, commuting services, and a mix of both, based on the median number of kinds of services used during the observation period. In-home services included home-help, bathing service, nursing visits, rental services for assistive devices and guidance conducted by doctors or other personnel. Commuting services included day care, outpatient rehabilitation and short stays for care/assistance in daily activities. If both in-home and commuting services were used, we considered this a mixed service. Dummy variables for each type of service were created, and mixed services were considered the reference.

The number of kinds of services was determined by the median number of services utilized during the survival time and was included in the model as a dichotomous variable; the use of two or more services was the group of interest, and the use of only “one service” was considered the reference.

The amount of services delivered was calculated as the total days on which services were utilized during the survival time divided by the number of months of survival to create a continuous time-dependent variable.

Age indicates age in years at enrollment in the study. Gender was a dichotomous variable, and female was chosen as the variable of interest. Insurer of City A classify insured income level, in 6 categories from level 1(the low) to level 6 (the high) according to taxation level of household members and/or elderly beneficiaries. Income level was included as a continuous variable. Length of stay in the LTCI system was included as a continuous variable to assess participant continuity in the LTCI system. Additionally, we calculated the utilization rate for insurance benefits (URB), i.e., the monthly proportion of insurance benefit units effectively used by a recipient divided by the fixed limits of benefits for CL1 in the LTCI system. The limit of benefits at CL1 was 16,580 units/month. URB was calculated for the overall time until the event of interest or until censoring occurred, and it was included in the model as a continuous variable.

To compare survival curves and evaluate the effect of in-home and community-based services on length-of-stay in CL1 among disability transition strata, we included a categorical variable containing disability transition status as “improved” or “declined” at the event time. Subjects who showed no change from the former CL1 during all observation periods were considered the reference group.

### Statistical analysis

We used Cox proportional-hazard regression analysis to model the effect of each covariate affecting the length of stay at CL1. The length of stay at CL1 (months) corresponded to “survival time” to assure a reasonable time to observe the occurrence of an event. The end of the observation period was set at 36 months after participants became LTC users. Subjects whose survival time exceeded 36 months were considered censored at 36 months (n = 37). However, because people in the lowest eligibility level are generally re-evaluated every 6 months by the insurer, we conducted a separate analysis every 6 months during the first 2 years of the observation period. The Kaplan–Meier method was used to obtain crude survival estimates, and survivor functions were plotted to compare disability transition strata.

As a first step in the Cox regression analysis, a univariate analysis of each predictor affecting the overall length of stay at CL1 by each phase of the observation period was investigated. The effect of transition disability status was also examined in the univariate analysis. Second, the effect of the covariates with potential confounders controlled was tested in each phase of the observation period through a single multivariate Cox regression analysis. Finally, separate hazard models were conducted to examine length of stay at CL1 across phases of the observation period for both the “improved” and “declined” disability transition strata. Age, gender and income level were entered as covariates in both strata analyses. Given the moderate sample size of these strata, a likelihood-ratio statistics was used to test the null hypothesis that all the coefficients associated with the covariates were zero.

Multicollinearity was examined via a correlation matrix and multicollinearity diagnostic statistics. A residual analysis to detect outliers and influential data was performed using deviance residual plots. Values of the deviance residual >2.5 were considered outliers and were excluded from the final analysis. The proportional-hazards assumptions were tested by including covariates by log-time interactions in the models. Goodness of fit of the models was evaluated as a function of the log-likelihood of the model with all parameter estimates and the log-likelihood of the model without the set of covariates. Data were analyzed with SAS software version 9.1 for Windows (SAS Institute, Inc., Cary, NC, USA).

## Results

### Descriptive analysis

Before the 2005 LTCI reform in Japan, people certified for CL1 benefits represented over 30% of those certified in the LTCI system [28]. In City A, subjects newly certified at CL1 (*n* = 529) represented 36% of all newcomers into the LTCI system during the accrual period of the study. Of these, 369 met the inclusion criteria of the study, accounting for 69.8% of all CL1 newcomers.

Table 1 summarizes the baseline characteristics of the study cohort by disability transition strata. The mean age of participants was 81 years old, and females represented 69% of the participants. Fifty-six percent of the participants were at income level 4, i.e., some household member is subject to taxation, but pension recipient is tax-free. A decline or an improvement in functional status, was observed in 43% and 27% of the sample, respectively. Thirty percent of the cohort remained at CL1 throughout the study period.

**Table 1:** Baseline characteristics of the study cohort by disability transition strata (n = 369)

**Variables**	**Improve (n = 99)**	**Equal (n = 112)**	**Decline (n = 158)**	**Total (n = 369)**
**Age (years) [mean,SD]**	**79** (7.5)	**80** (7.3)	**82** (7.8)	**81** (7.7)
**Female [n, (%)]**	**72 (73%)**	**79 (71%)**	**103 (65%)**	**254 (69%)**
**Income level [n, (%)]**				
**level 1**	**6 (6%)**	**3 (3%)**	**4 (3%)**	**13 (4%)**
**level 2**	**26 (26%)**	**17 (15%)**	**24 (15%)**	**67 (18%)**
**level 3**	**11 (11%)**	**19 (17%)**	**20 (13%)**	**50 (14%)**
**level 4**	**47 (47%)**	**62 (55%)**	**98 (62%)**	**207 (56%)**
**level 5**	**4 (4%)**	**5 (4%)**	**7 (4%)**	**16 (4%)**
**level 6**	**5 (5%)**	**6 (5%)**	**5 (3%)**	**16 (4%)**
**Type of LTC Services [n, (%)]**				
**commuting services**	**42 (43%)**	**57 (51%)**	**81 (51%)**	**180 (49%)**
**in-home services**	**34 (34%)**	**32 (29%)**	**34 (22%)**	**100 (27%)**
**mixed**	**23 (23%)**	**23 (20%)**	**43 (27%)**	**89 (24%)**
**Number of LTC Services (monthly) [n, (%)]**				
**1 service**	**67 (68%)**	**78 (70%)**	**88 (56%)**	**233 (63%)**
**2 services**	**24 (24%)**	**27 (24%)**	**55 (35%)**	**106 (29%)**
**3+ services**	**8 (8%)**	**7 (6%)**	**15 (9%)**	**30 (8%)**
**Utilization of LTC Services(days/month) [mean,SD]**				
**commuting services**	**6.9** (6.23)	**8.4** (5.26)	**9.3** (4.35)	**8.5** (5.18)
**in-home services**	**18.4** (11.12)	**19.2** (11.86)	**24.2** (18.36)	**20.7** (14.30)
**mixed**	**27.2** (13.23)	**26.6** (11.34)	**25.3** (12.08)	**26.1** (12.09)
**Utilization Rate of Benefits [mean,SD]**	**0.324** (0.188)	**0.361** (0.229)	**0.472** (0.228)	**0.399** (0.228)
**Length-of stay in LTCI system (months)**				
**(mean, [SD])**	**40** (17.32)	**29** (16.82)	**38** (17.06)	**36** (17.62)
**(median; [min,max])**	**39** (9–72)	**26** (6–69)	**37** (6–72)	**34** (6–72)
**Length-of stay in Care Level 1 (months)**				
**(mean, [SD])**	**17** (13.83)	**21** (12.93)	**14** ( 10.93)	**17** (12.72)
**(median, [ min,max])**	**12** (3–61)	**19** (5–61)	**10** ( 3–48)	**14** (3–61)

Forty-nine percent of participants used commuting services an average of 8.5 days/month. In-home services were used by 27% of the participants at an average rate of 20.7 days/months. In total, 63% of the subjects used only one service. The mean URB in the cohort was 0.399. The median length of stay in the LTCI system was 34 months and the median length of stay at CL1 was 14 months.

### Survival analysis

**Figure 1:**
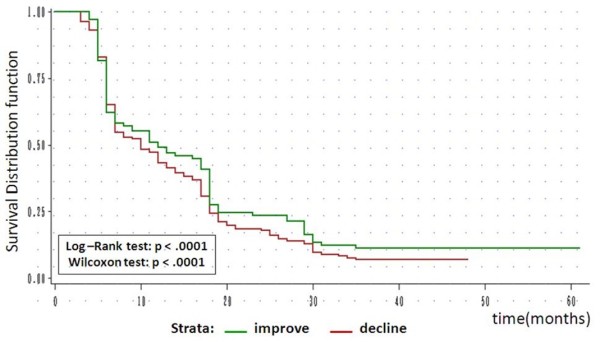
Survivor functions by disability transition strata (n = 369).

Figure 1 shows the survivor functions for the disability transition strata. After 6 months, subjects whose functional status improved had a significantly longer stay at CL1 than did participants whose functional status declined.

Kaplan–Meier estimates for the overall data showed that the estimated probability of a subject’s remaining at CL1 for 6 months or more was 72%, the probability of remaining for 12 months or more was 59%, that for 18 months or more was 43%, that for 2 years or more was 38%, and that for 3 years or more was 24%. The median duration of stay at CL1 was 18 months for censored cases and 9 months for uncensored cases (data not shown).

### Cox proportional-hazards models

In the univariate analysis, none of the potential predictors was statistically significant across phases of the observation period. However, the long-rank test of equality for disability transition strata was highly significant. Hazard ratios for the “improved” disability transition strata showed a tendency to decline across phases of the observation period. Conversely, a trend toward an increase in the relative risk across phases of the observation period was observed for the “declined” transition (data not shown).

Moderate and expected associations between the use of two or more services and commuting services (0.59) and the use of two or more services and in-home services (0.57) were observed in the correlation matrix. The variance inflation factor (VIF) values for the covariates ranged from 1.02 to 2.18, indicating no multicollinearity. The residual analysis of the Cox regression model for disability strata detected two observations (one in each stratum analysis) that affected model fit; thus, they were considered outliers and removed from the final analysis. The tests of all time-dependent variables were not significant individually or collectively in both the “declined” disability strata (*p* = 0.42) and the “improved” disability strata ( *p* = 0.75), so the assumption of model proportionality was fulfilled.

After controlling for potential confounders across all phases of the observation period, an overall multivariate Cox regression analysis showed that only the amount of services delivered at 36 months had a significant effect on the probability of a change from CL1. Thus, for each 1 day increase in the amount of monthly services utilized, the probability of change from CL1 dropped by an estimated 1.6% (data not shown).

### “Improved” disability transition stratum

In this stratum, the estimated probabilities that a subject would stay at CL1 for 6 months or more was 62%, the probability that the subject would stay for 1 year or more was 49%, for 18 months or more was 28%, for 2 years or more was 24%, and at 35 months, the longest time to censoring was 11%. The median length of stay at CL1 was 12 months (95% confidence interval [CI], 7–18 months).

Table 2 shows the results of the Cox regression model in this stratum. Women had a significantly greater probability of improving their functional status during all phases of the observation period than did men, but the rate of improvement decreased across time. Age and income level were not significant during all phases of the observation period. After controlling for other variables, the use of two or more services was marginally associated (*p*-value < 0.10) with a decrease (from 72% to 60%) in the probability of improving one’s functional status after 12 months of stay in the system. Taking the reciprocal, users of only “one service” had a 3.6 times greater chance of improving their functional status than did users of two or more services at 12 months into the observation period. Moreover, a marginal effect (*p* = 0.09) at 12 months was observed for the amount of services delivered. Thus, for each 1-day increase in the amount of services used, the possibility of improving one’s functional status increased by an estimated 3%.

**Table 2:** Multivariate hazard ratios and 95% CIs for covariates by phases of the observation period in Improve functional status strata (n = 210)

**Models**	**6-months**			**12-months**			**18-months**			**24-months**			**36-months**		
	**Hazard ratio**	**95% CI**	***p-value***	**Hazard ratio**	**95% CI**	***p-value***	**Hazard ratio**	**95% CI**	***p-value***	**Hazard ratio**	**95% CI**	***p-value***	**Hazard ratio**	**95% CI**	***p-value***
**Age**	**0.99**	0.94 - 1.04	0.653	**0.98**	0.94 - 1.03	0.400	**1.00**	0.97 - 1.04	0.902	**1.01**	0.97 - 1.04	0.758	**1.01**	0.98 - 1.05	0.495
**Gender**															
**Male**	**1.00**			**1.00**			**1.00**			**1.00**			**1.00**		
**Female**	**4.51**	1.51 - 13.50	0.007	**3.32**	1.47 - 7.50	0.004	**1.74**	0.95 - 3.17	0.072	**1.68**	0.94 - 3.01	0.082	**1.84**	1.06 - 3.21	0.031
**Income level**	**0.95**	0.68 -1.33	0.764	**0.97**	0.74 - 1.28	0.840	**0.87**	0.70 - 1.09	0.218	**0.84**	0.68 - 1.04	0.113	**0.89**	0.73 - 1.09	0.251
**Length of stay in LTCI system**	**0.96**	0.94 - 0.98	0.001	**0.97**	0.95 - 0.99	0.001	**0.96**	0.95 - 0.98	<.0001	**0.96**	0.95 - 0.98	<.0001	**0.96**	0.95 - 0.98	<.0001
**Utilization Rate of Benefits**	**0.24**	0.02 - 2.68	0.245	**0.14**	0.02 - 1.10	0.061	**0.24**	0.05 - 1.23	0.087	**0.34**	0.07 - 1.65	0.182	**0.48**	0.11 - 2.10	0.332
**Number of LTC Services**															
**1 service**	**1.00**			**1.00**			**1.00**			**1.00**			**1.00**		
**2 + services**	**0.41**	0.08 - 2.04	0.274	**0.28**	0.07 - 1.08	0.066	**0.37**	0.12 - 1.12	0.079	**0.35**	0.12 - 1.08	0.067	**0.40**	0.14 - 1.12	0.081
**Type of LTC Services**															
**mixed**	**1.00**			**1.00**			**1.00**			**1.00**			**1.00**		
**commuting services**	**0.46**	0.08 - 2.57	0.376	**0.44**	0.10 - 1.86	0.264	**0.45**	0.15 - 1.40	0.169	**0.48**	0.16 - 1.44	0.189	**0.46**	0.17 - 1.27	0.133
**in-home services**	**0.28**	0.06 - 1.42	0.124	**0.35**	0.09 - 1.43	0.145	**0.39**	0.13 - 1.20	0.100	**0.38**	0.13 - 1.17	0.093	**0.41**	0.15 - 1.14	0.087
**Amount of LTC Services (days/month)**	**1.02**	0.97 - 1.06	0.457	**1.03**	1.00 - 1.07	0.090	**1.02**	0.98 - 1.05	0.382	**1.01**	0.98 - 1.05	0.469	**1.01**	0.97 - 1.04	0.779
**n / censored**	**210/173**			**210/160**			**210/139**			**210/135**			**210/123**		
**Likelihood ratio test**	0.002			0.002			0.0011			0.001			<.0001		

### “Declined” disability transition stratum

In this stratum, Kaplan–Meier estimates showed that the estimated probabilities that a subject would stay at CL1 for 6 months or more was 65%, the probability of staying for 1 year or more was 43%, for 18 months or more was 24%, for 2 years or more was 18%, and for 35 months, the longest time to censoring, it was 7%. The median duration of stay at CL1 for those in the “declined” stratum was 10 months (95% CI, 7–13 months).

Table 3 shows the results of the Cox regression model in this stratum. Despite the adequacy of the models across all the observation phases, and with an exceptionally marginal effect (*p*-value around 0.10) for the amount of services at 18-months and after, none of the remaining covariates was significantly associated with the hazard ratio for a decline in functional status. As the hazard ratio was 0.98 for the amount of LTC services, this means that for each day of added services used, the probability of decline in functional status decreased by an estimated 2%.

**Table 3:** Multivariate hazard ratios and 95% CIs for covariates by phases of the observation period in Decline functional status strata ( n = 269)

**Models**	**6-months**			**12-months**			**18-months**			**24-months**			**36-months**		
	**Hazard ratio**	**95% CI**	***p-value***	**Hazard ratio**	**95% CI**	***p-value***	**Hazard ratio**	**95% CI**	***p-value***	**Hazard ratio**	**95% CI**	***p-value***	**Hazard ratio**	**95% CI**	***p-value***
**Age**	**1.00**	0.97 - 1.04	0.895	**1.01**	0.98 - 1.04	0.673	**1.00**	0.97 - 1.02	0.854	**1.00**	0.98 - 1.02	0.880	**1.00**	0.98 - 1.03	0.791
**Gender**															
**Male**	**1.00**			**1.00**			**1.00**			**1.00**			**1.00**		
**Female**	**0.93**	0.53 - 1.64	0.800	**0.95**	0.60 - 1.49	0.820	**1.05**	0.70 - 1.57	0.808	**1.12**	0.76 - 1.66	0.560	**1.01**	0.70 - 1.45	0.963
**Income level**	**0.99**	0.73 - 1.34	0.933	**0.86**	0.68 - 1.10	0.228	**0.91**	0.73 -1.12	0.364	**0.92**	0.75 - 11.3	0.417	**0.88**	0.72 - 1.07	0.201
**Length of stay in LTCI system**	**0.95**	0.93 - 0.97	<.0001	**0.97**	0.95 - 0.98	<.0001	**0.96**	0.95 - 0.98	<.0001	**0.96**	0.95 - 0.98	<.0001	**0.96**	0.95 - 0.97	<.0001
**Utilization Rate of Benefits**	**0.91**	0.20 - 4.08	0.898	**1.04**	0.35 - 3.10	0.946	**1.04**	0.40 - 2.73	0.930	**1.24**	0.49 - 3.10	0.650	**1.30**	0.54 - 3.14	0.562
**Number of LTC Services**															
**1 service**	**1.00**			**1.00**			**1.00**			**1.00**			**1.00**		
**2 + services**	**1.39**	0.63 - 3.08	0.419	**1.17**	0.61 - 2.25	0.643	**1.25**	0.71 - 2.20	0.440	**1.15**	0.67 - 2.00	0.610	**1.13**	0.67 - 1.91	0.647
**Type of LTC Services**															
**Mixed**	**1.00**			**1.00**			**1.00**			**1.00**			**1.00**		
**commuting services**	**1.17**	0.46 - 2.98	0.736	**1.14**	0.55 - 2.35	0.722	**1.04**	0.55 - 1.94	0.915	**0.95**	0.52 - 1.74	0.865	**0.99**	0.56 - 1.76	0.975
**in-home services**	**0.71**	0.27 - 1.92	0.505	**1.00**	0.48 - 2.08	0.994	**0.98**	0.51 - 1.89	0.957	**0.91**	0.48 - 1.70	0.761	**1.00**	0.55 - 1.83	0.993
**Amount of LTC Services (days/month)**	**0.98**	0.95 - 1.02	0.303	**0.99**	0.97 - 1.02	0.622	**0.98**	0.96 - 1.00	0.092	**0.98**	0.96 - 1.00	0.120	**0.98**	0.96 - 1.00	0.085
**n / censored**	**269/214**			**269/180**			**269/150**			**269/140**			**269/123**		
**Likelihood ratio test**	<.0001			0.007			<.0001			<.0001			<.0001		

## Discussion

The LTCI system was implemented (April 2000) to prevent a decline in functional status and allow the elderly to live independently in their homes as long as possible, but it has become an important issue in Japan. In the last decade, studies on the effects of LTC services have mainly focused on disability transition [20-22]. or on beneficiaries’ risk of hospitalization or institutionalization [22,27]. In these studies, scale-up in LTCI eligibility levels was a valid response to the decline in functional status, and hospitalization or institutionalization were considered adverse events.

An important concern in studies related to the effect of LTC services on the progression of disability in LTCI users has been the difficulty of adjusting for individual medical conditions [22]. A partial explanation is that data for the LTCI system are recorded from an insurer’s perspective, so data about medical conditions are absent. As the effect of medical condition could be a confounder, we tried to minimize this effect on the progression of disability. We did this, first, by focusing on mildly impaired subjects, who were assumed to have better health status than the average of all elderly users of the LTCI system, and second, by ruling out the probability of hospitalization episodes requiring “continuity” in the utilization of LTC services, as explicitly mentioned in the inclusion criteria.

Tomita et al. suggested that in-home and community-based services contribute to encouraging individuals to live independently at home as long as possible [22]. Kato et al. concluded that respite stay in a nursing home and the use of additional services are associated with a decline in the functional status of users with a lower care needs levels [21]. Ishibashi et al. demonstrated that home-help service users have a lower risk of functional decline than do day-care services users, and providing more home-help services did not lead to a greater decline in functional status [19].

In our study, we demonstrated a significant gender difference in favor of women across all phases of the observation period, but it decreased over time in those whose functional status improved. Additionally, the probability of improving one’s functional status at 12 months was marginally associated with an increased use of services (day/months) and with the use of only “one service”. Based on the assumption that in-home and community-based services are effective if the amount and mix of services delivered are adequate, two possible mechanisms may explain the weak effects of in-home and community-based services on disability transition in our study. First, the change in care-needs level as a measure of disability progression may not be specific enough to assess changes in functional status derived from the LTC services supplied. Second, in-home and community-based services provided in City A may be inappropriate in terms of intensity and duration or quality of care, as just another possible explanation.

## Conclusion

In conclusion, the observed effects of in-home and community-based services on disability transition status of CL1 newcomers were considered fairly modest and inconsistent in terms of their ability to improve or to prevent a decline in functional status of this LTCI system cohort in City A. These findings must be interesting for local insurer considering that CL1 current users they are using less than 40% of the LTCI benefits to which they are entitled. Finally, these findings suggest two possibilities. First, there may be a need to increase the amount of services and/or change the mixture of services delivered. Alternatively, it may be necessary to take appropriate measures to assess the effectiveness and cost-effectiveness of these services.

Some study limitations should be considered. Although the study population was proportionately representative of the elderly population in Japan, the study was limited to a small suburban area of Tokyo. The possibility that our results were affected by other social factors, such as living arrangements or informal care, which have been reported to have a significant impact on functional status [29-32] cannot be ruled out entirely. Therefore, these results must be confirmed in a large-population-based survey, ideally a randomized controlled trial, with control of possible confounders such as demographic, social, medical, and insurance factors to investigate the causal relationship between LTC care-services utilization and the progression of disability in the Japanese elderly population.

## Competing interests

The authors declare that they have no competing interests.

## Authors' contributions

PO-T carried out structuring the study design, statistical analysis, interpreting the data, and drafting the manuscript. NT supervised all the process as the corresponding author: participated in the design of the study, acquiring the data, interpretation of the data, and helped to finalize the manuscript.MK participated in designing this study, acquiring the data, and structuring the data set. All authors read and approved the final manuscript.

## Pre-publication history

The pre-publication history for this paper can be accessed here:

http://www.biomedcentral.com/1472-6963/12/239/prepub

## Supplementary Material

Additional file 1 **Annex 1.** Frequency distribution of LTC services delivered to a cohort of CL1 users in LTCI system in Japan. Click here for file
