# Supplementary material for: Effect of in-home and community-based services on the functional status of elderly in the long-term care insurance system in Japan
Source: BMC Health Serv Res. 2012 Aug 4;12:239. doi: 10.1186/1472-6963-12-239 (PMC3505459; doi:10.1186/1472-6963-12-239)
Supplement: Additional file 1 — Annex 1. Frequency distribution of LTC services delivered to a cohort of CL1 users in LTCI system in Japan. [file 1472-6963-12-239-S1.doc]

**Annex 1: Frequency distribution of LTC services delivered to a cohort of CL1 users in LTCI system in Japan.**
